# How to Cure a Cold

**Published:** 1870-10

**Authors:** 


### HOW TO CURE A COLD.

The following is from a lecture by Dr. G. Johnson, the Professor of Medicine in King's College:—

The exciting cause of a catarrh, in the great majority of cases, is a chill, or some unknown atmospheric influence, which tends to suppress the action of the skin. The popular treatment consists in the use of a hot foot-bath at bed-time, a fire in the bedroom, a warm bed, and some hot drink taken after getting into bed, the diaphoretic action being assisted by an extra amount of bedclothes. Complete immersion in a warm bath is more efficacious than a foot-bath; but the free action of the skin is much more certainly obtained by the influence of hot air,—most surely and profusely, perhaps, by the Turkish bath. The Turkish bath, however, is not always to be had, and even when available, its use in the treatment of catarrh is attended with some inconvenience. In particular, there is the risk of a too speedy check to the perspiration after the patient leaves the bath. On the whole, the plan which combines in the greatest degree efficiency with universal applicability consists in the use of a simple hot-air bath, which the patient can have in his own bedroom. All that is required is a spirit lamp with a sufficiently large wick. Such lamps are made of tin, and sold by most surgical instrument makers.

The lamp should hold sufficient spirits to burn for half an hour. The patient sits undressed, in a chair, with the lamp between his feet, rather than under the chair. An attendant then takes two or three blankets and folds them round the patient from his neck to the floor so as to enclose him and the lamp, the hot air from which passes freely round his body. In from a quarter to half an hour there is usually a free perspiration, which may be kept up for a time by getting into bed between hot blankets. I have myself gone into a hot air bath suffering from headache, pain in the limbs, and other indications of a severe incipient catarrh, and in the course of half an hour I have been entirely and permanently freed from these symptoms by the action of the bath.

Another simple and efficient mode of exciting the action of the skin consists in wrapping the undressed patient in a sheet wrung out of warm water, then, over this, folding two or three blankets. The patient may remain thus "packed" for an hour or two, until free perspiration has been excited. Let me impress upon you that the sweating plan of treatment, to be successful in cutting short the disease, must be adopted early,—I mean within a few hours from the commencement of the symptoms.—*British Medical Journal.*

